# Association of the CD28 markers with the disease activity in systemic lupus erythematosus patients

**DOI:** 10.12688/f1000research.140890.1

**Published:** 2023-10-18

**Authors:** Mirza Zaka Pratama, Kusworini Handono, Handono Kalim, Hani Susianti

**Affiliations:** 1 Internal Medicine, Universitas Brawijaya, Malang, East Java, Indonesia; 2 Clinical Pathology, Universitas Brawijaya, Malang, East Java, Indonesia

**Keywords:** Systemic Lupus Erythematosus, CD28 markers, disease monitoring

## Abstract

**Background:** Systemic Lupus Erythematosus (SLE) is a complex autoimmune disease with diverse manifestations and unpredictable activity. CD28 markers, particularly sCD28, is a promising biomarker for evaluating SLE disease activity. This study aimed to investigate the significance of CD28 markers in evaluating disease activity in SLE and the role of sCD28 in various clinical manifestations.

**Methods:** A total of 40 female subjects, aged between 18 and 45 years, who fulfilled the 2019 EULAR/ACR classification criteria for SLE were recruited in this study. Twenty healthy matched individuals were also recruited as control. Comprehensive data on demographic information, clinical manifestations, laboratory test findings, and treatment history were collected from all participants. The Indonesian version of SLEDAI-2K score was utilized to assess disease activity, categorizing patients into active SLE and lupus low disease activity (LLDAS). Collected data were analyzed on SPSS for Windows version 25.0.

**Results:** Patients with SLE in LLDAS category had significantly lower SLEDAI scores (1.8 ± 1.4 vs 11.7 ± 4.9, p<0.001) with mild clinical manifestation. Active SLE patients had the lowest percentages of CD4 ^+^CD28 ^+^ cells (5.7 ± 4.1%) and the highest sCD28 concentration (26.2 ± 11.3 ng/ml) compared to other groups. Moreover, sCD28 concentration demonstrated a moderate positive correlation with SLE disease activity. In most cases, higher sCD28 concentrations were associated with clinical manifestations, particularly in neuropsychiatric lupus (OR 7.1 [1.8 – 67.9], p=0.047), nephritis (OR 14.5 [1.6 – 131.9], p=0.017), and mucocutaneous manifestations (OR 3.4 [1.9 – 12.8], p=0.035).

**Conclusions:** Our study establishes the link between CD28 markers and disease activity, including certain clinical manifestations in SLE. We suggest that CD28 has a potential role in predicting disease activity. However, further research through longitudinal studies is required to strengthen these findings.

## Introduction

Systemic Lupus Erythematosus (SLE) is an autoimmune disease predominantly in women, leading to inflammation and damage in various organs and tissues. The clinical manifestations and disease course of SLE can vary widely among patients, making it challenging to predict disease activity and tailor appropriate treatment strategies. ^1^ In recent years, researchers have been investigating potential biomarkers that could aid in the assessment and management of SLE. One promising biomarker is the Cluster of Differentiation 28 (CD28) marker. ^2^^–^^4^ CD28 is a surface co-stimulatory molecule in T-cells, playing a crucial role in regulating T-cell activation and proliferation. It interacts with its ligands on antigen presenting cells, CD80 and CD86, to facilitate effective T-cell responses. ^5^ In certain conditions, CD28 can be cleaved under certain conditions from the T-cell surface, releasing soluble CD28 into the bloodstream. ^6^ The soluble form of CD28, referred to as sCD28, has attracted much interest as a potential biomarker for autoimmune disorders, such as SLE. ^7^

Our previous study observed a reduction in CD28 expression on T-cell surfaces, which was linked to comorbidities in patients with SLE. ^8^ This downregulation of CD28 may impair the normal regulatory functions of T-cells, leading to uncontrolled immune responses and increased disease activity. ^9^^,^^10^ The levels of sCD28 in the blood of patients with SLE or other autoimmune disorders are higher than those of healthy individuals as shown in previous studies. ^11^^,^^12^ Increased sCD28 levels may contribute to the autoimmune response’s persistence in SLE. ^13^

However, the clinical presentation of SLE can vary widely among patients, suggesting the existence of different phenotypes with distinct underlying pathogenic mechanisms. ^14^ Little studies have described the role of the sCD28 markers according to the disease phenotypes in SLE patients. Therefore, a study is needed to identify the mechanisms and substantiate the clinical efficacy of soluble CD28 as a biomarker in SLE. Thus, this study is going to discover the role and performance of CD28 markers in assessing the disease activity in SLE. We also aimed to observe the role of sCD28 markers in different clinical manifestations in SLE patients. The investigation of sCD28 as a biomarker in SLE may hold promise for improving the diagnosis, assessment, and management of this complex autoimmune disease.

## Methods

### Study populations

The subjects in this study consisted of forty patients diagnosed with SLE. Patients were recruited with the consecutive sampling from the Rheumatology Clinic at Saiful Anwar General Hospital in Malang, Indonesia, throughout the designated study period (March to December 2020). The study participants consisted exclusively of female individuals aged between 18 and 45 who met the 2019 European League Against Rheumatism/American College of Rheumatology (EULAR/ACR) categorization criteria for SLE. Briefly, patients should have a positive ANA as a mandatory entry criterion, followed by additional criteria that were scored from 2 to 10 (constitutional, hematological, neuropsychiatric, mucocutaneous, serosal, musculoskeletal, renal, antiphospholipid antibodies, complement proteins, and SLE-specific antibodies). Patients with score ≥10 points would be classified as SLE. ^15^ We excluded pregnant or breastfeeding patients with ongoing infection or malignancy. Twenty individuals in healthy condition, matched in age and gender, were chosen as control subjects. Demographic data, clinical symptoms, laboratory test findings, and therapeutic history were reported for all individuals.

### Ethical considerations

This study followed the Helsinki Declaration and the ethical committee of Saiful Anwar General Hospital in Malang, Indonesia, granted its approval for this research (ethical number approval 400/085/K.3/302/2020, approved on 23 ^rd^ March 2020). Written informed consent was also obtained from all participants involved.

### SLE disease activity assessment

The patients’ disease activity was evaluated using the Systemic Lupus Erythematosus Disease Activity Index 2000 (SLEDAI-2K). ^16^ The SLEDAI-2K score was evaluated by a rheumatologist in the Rheumatology Clinics. Routine laboratory tests, such as complete blood count, urinalysis, anti-dsDNA, C3, and C4 were conducted as part of the SLEDAI-2K assessment. The radiologic and biopsy examinations were also performed as indicated according to the disease manifestations. SLE patients were classified according to the disease activity: active and lupus low disease activity state (LLDAS) SLE patients. LLDAS was categorized based on the previous study. ^17^

### Flowcytometry examination

The flowcytometry examination was used to measure the percentages of membrane or surface CD28 expression within the CD4 and CD8 T-cell subsets. The investigation involved the evaluation of CD28 membrane expression percentages in peripheral blood mononuclear cells (PBMC) from subjects. A venous blood sample of 10-15 cc was obtained from each participant. PBMCs were extracted from using Lymphoprep (Stemcell Technology) through centrifugation. The produced PBMC layer was systematically eliminated and washed twice using 10 cc of phosphate-buffered saline (PBS). Afterward, the supernatant was eliminated, and the remaining supernatant was centrifuged at room temperature. FITC anti-human CD4, PerCP anti-human CD8, and PE anti-human CD28 (Biolegend) was used as the staining. The percentages of CD4 ^+^CD28 ^+^ and CD8 ^+^CD28 ^+^ cells were assessed using flow cytometry (BD FACScalibur). The measurements were performed on 10,000 cells. The results were expressed as percentages (%) of cells.

### Enzyme-Linked Immunosorbent Assay (ELISA) examination

The ELISA examination was performed to measure the concentration of serum sCD28 markers. The analysis of serum SCD28 was performed using the ELISA kit provided by Bioassay Technology Laboratory (cat number E4196hu), following the methods recommended by the manufacturer. Briefly, serum collected from the blood of the subjects by centrifugation at 2000-3000 rpm for 30 minutes. Samples and ELISA reagents were added into each well and incubated for 1 hour at 37 ^o^C. The plate was washed five times and the substrate solution were added. Samples were incubated for 10 minutes at 37 ^o^C, then the stop solution were added. Samples were read the optical density (OD) value within 10 minutes. The sCD28 marker levels were measured in nanograms per milliliter (ng/ml).

### Statistical analysis

Normally distributed data were characterized using mean and standard deviation, while skewed data were represented by median and interquartile range. Categorical data were presented as percentages. Unpaired t-test or Mann-Whitney test was utilized to compare numerical data between groups. The Chi-square or Fisher exact test was employed to conduct a comparative analysis of two sets of categorical data. Pearson’s correlation was used to examine the relationships between each senescence marker and the SLEDAI score. Receiver operating characteristic (ROC) curves were applied to distinguish between active and LLDAS SLE. Logistic regression models were used to evaluate the association between the variables. The statistical significance of the results was determined based on a p-value of <0.05. Data analysis was conducted in SPSS for Windows version 25.0.

## Results

### Subject characteristics

There are twenty healthy individuals, as well as forty SLE patients. The patients with SLE were categorized into two groups: active SLE patients (n=20) and SLE patients in low disease activity state (LLDAS) (n=20). The characteristics of the subjects are displayed in Table 1. All subjects were females of similar ages. Patients with Lupus Low Disease Activity State (LLDAS) showed notably lower SLEDAI score than individuals with active SLE (1.8 ± 1.4 vs 11.7 ± 4.9, p<0.001). The clinical manifestations of SLE patients in both groups are presented in Table 1. Patients with LLDAS showed mild manifestations, such as mucocutaneous lesions or hematologic issues, but none had hemolytic anemia. The anti-dsDNA levels of the active SLE patients were markedly higher compared to LLDAS patients (89.2 ± 62.4 vs. 51.8 ± 46.7 IU/ml, p=0.038).

**Table 1.** Characteristic of the subjects.

Variables	Healthy individuals (n=20)	LLDAS SLE patients (n=20)	Active SLE patients (n=20)	p
Age (years old)	33.5 ± 2.2	34.0 ± 13.3	30.1 ± 9.8	0.304
SLEDAI score	-	1.8 ± 1.4	11.7 ± 4.9	<0.001
Clinical manifestations, n (%)				
- Neuropsychiatric	-	0 (0)	6 (30)	0.008
- Vasculitis	-	0 (0)	3 (15)	0.072
- Arthritis	-	0 (0)	6 (30)	0.008
- Myositis	-	0 (0)	5 (25)	0.017
- Nephritis	-	0 (0)	9 (45)	0.001
- Mucocutaneous	-	8 (40)	9 (45)	0.749
- Serositis	-	0 (0)	7 (35)	0.004
- Hematologic	-	9 (45)	13 (65)	0.204
Anti-dsDNA levels (IU/ml)	-	51.8 ± 46.7	89.2 ± 62.4	0.038

### Comparison of the membrane and soluble CD28 markers among subjects

**Figure 1.**
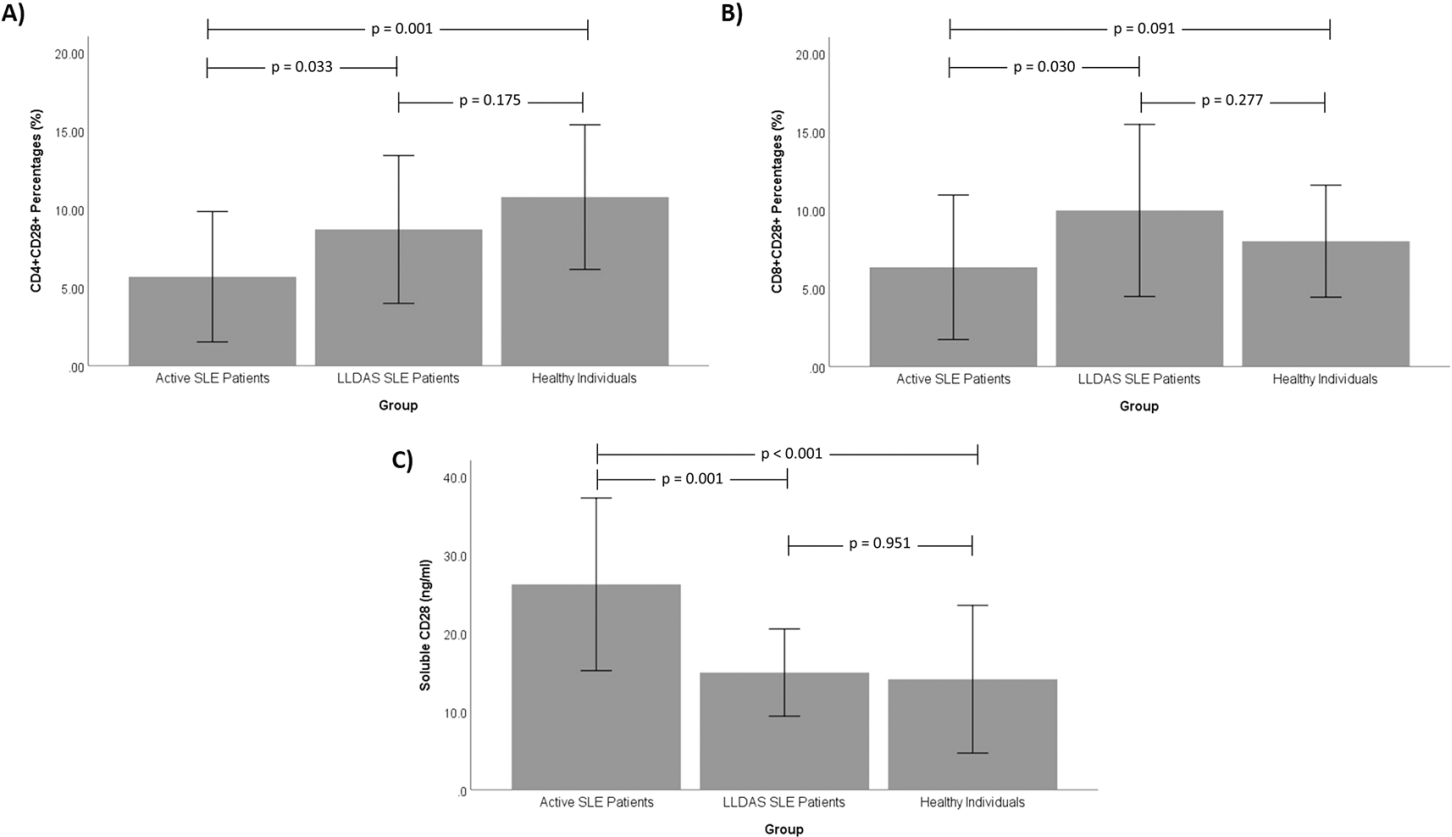
Comparison of the membrane and soluble CD28 concentrations between groups.

The percentages of membrane CD28 seen in both CD4+ and CD8+ T-cells were displayed in Figure 1. Among all groups, active SLE patients showed the lowest percentages of CD4+CD28+ (5.7 ± 4.1%). Notably, the percentages of CD4+CD28+ were significantly higher in LLDAS SLE patients (8.7 ± 4.7%, p=0.033) and healthy individuals (10.8 ± 4.6%, p=0.001) compared to active SLE patients. There was no statistically significant disparity was observed in the percentages of CD4+CD28+ cells between the patients with LLDAS SLE and the control group of healthy individuals (p=0.175). As for the CD8 ^+^ T-cell subset, the lowest percentages were found in active SLE patients (6.3 ± 4.6%) and statistically significant compared to LLDAS SLE patients (10.0 ± 5.5%, p=0.030). However, no significant differences were found in the CD8 ^+^CD28 ^+^ percentages between the SLE patients (both LDAS patients) with healthy individuals (8.0 ± 3.6%). The comparison of sCD28 between groups are also described in Figure 1. Among all groups, it was shown that active SLE patients had the highest concentration of sCD28 at 26.2 ± 11.3 ng/ml, which was found to be considerably higher when compared to both LLDAS patients with a value of 15.0 ± 5.6 ng/ml (p=0.001), as well as healthy individuals with a value of 14.1 ± 9.4 ng/ml (p<0.001).

### Correlation of the CD28 markers with the disease activity among patients

**Figure 2.**
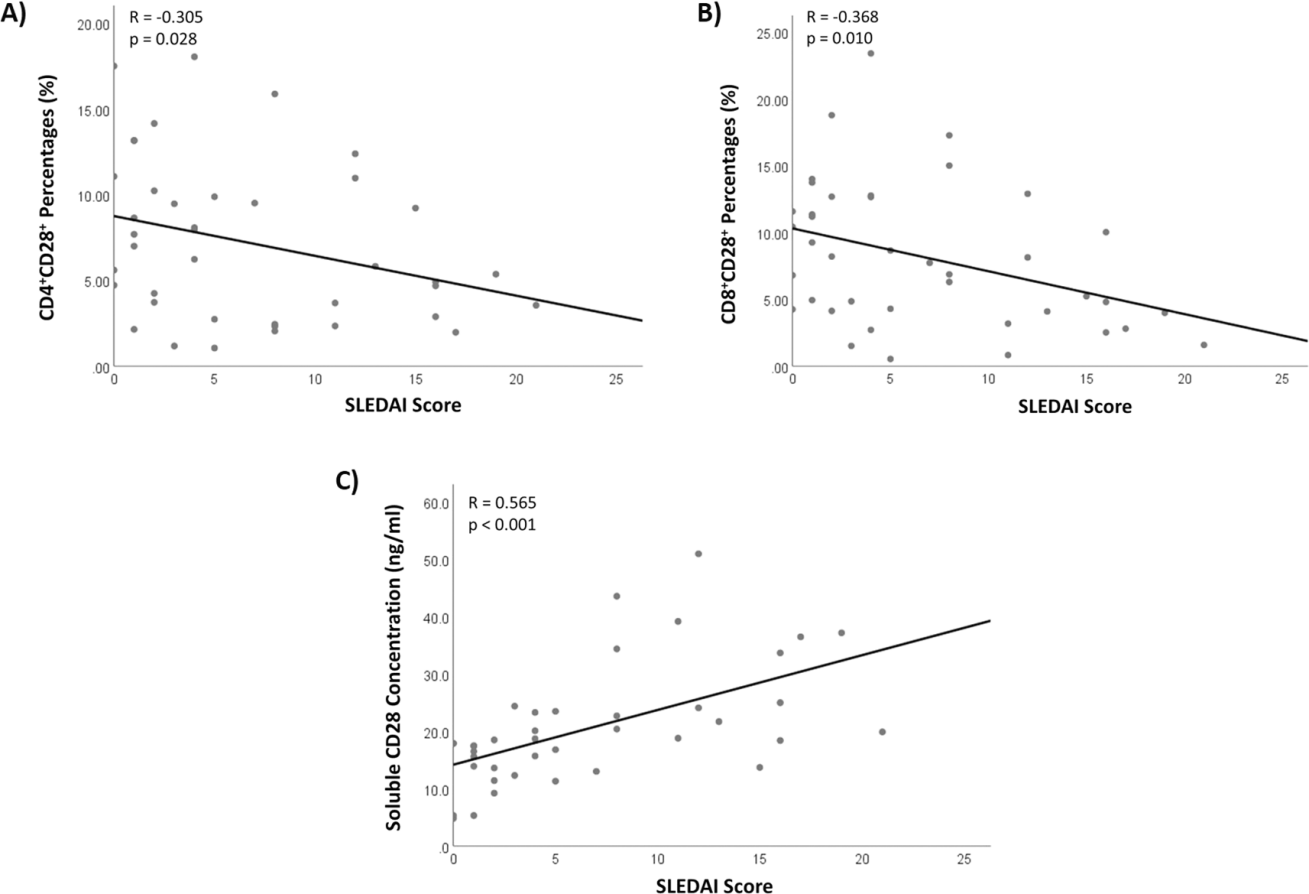
Correlation of the CD28 molecules levels with the disease activity.

The Pearson correlation analysis was conducted to evaluate the correlation between the CD28 markers and disease activity among the patient, as shown in Figure 2. The study revealed a weak negative correlation between the percentages of CD4+CD28+ cells and the SLEDAI score in SLE patients. Furthermore, a comparable correlation was observed between the percentages of CD8+CD28+ cells and the SLEDAI score. Conversely, the concentration of sCD28 exhibited a moderate positive correlation with the SLEDAI score.

**Figure 3.**
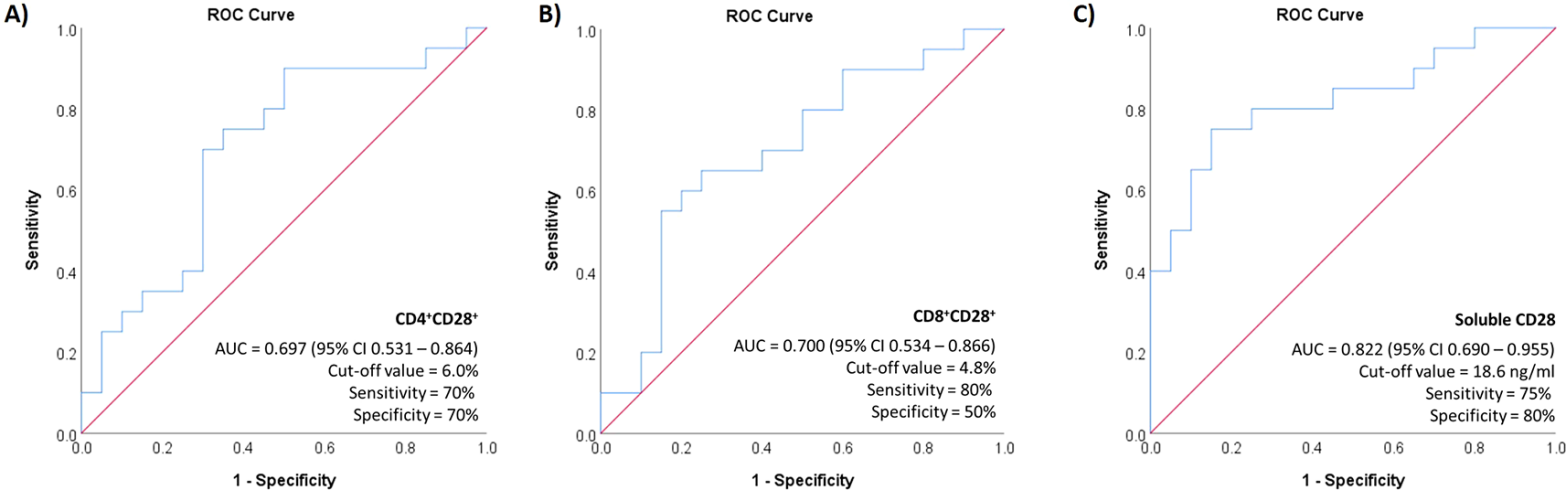
ROC curve analysis for the performance of CD28 markers in predicting the disease activity.

The study conducted ROC curve analysis to evaluate the performance of the CD28 marker in distinguishing between active SLE patients and SLE patients in low disease activity state (LLDAS), as depicted in Figure 3. The CD4+CD28+ had a relatively low AUC score, measuring 0.697 (95% CI 0.531 – 0.864) with a p-value of 0.033. Both the sensitivity and specificity were 70% for the cut-off value of 6.0%. We reported a greater AUC score for CD8+CD28+ percentages in the differentiation of active SLE patients (AUC 0.700 [95% CI 0.534 – 0.866], p=0.030). Using a cut-off value of 4.8%, the percentages of CD8+CD28+ exhibited a sensitivity of 80% and a specificity of 50%. The sCD28 had the highest AUC score of 0.822 (95% CI 0.690 – 0.955, p=0.001), demonstrating a sensitivity of 70% and specificity of 80% for a cut-off value of 18.6 ng/ml. We also conducted a comparison to evaluate the performance of anti-dsDNA as a conventional marker to differentiate between active and LLDAS SLE patients. The AUC score for anti-dsDNA was determined to be 0.740 (95% CI [0.585 – 0.895], p=0.020), along with the sensitivity and specificity of the test at a cut-off value of 85 IU/ml were found to be 40% and 80% respectively.

### Association of the soluble CD28 markers according to the clinical manifestations

SLE patients might have different phenotypes with various clinical manifestations. In order to observe the association between CD28 markers and disease phenotypes, a comparison of sCD28 markers among SLE patients based on their clinical manifestations was performed and presented in Table 2. In most cases, the sCD28 concentrations were higher in subjects with clinical manifestations. Nevertheless, notable differences were noted among patients with SLE who exhibited neuropsychiatric manifestations (p=0.031), nephritis (p=0.043), and serositis (p=0.007). The cut-off value was used to analyze the association between the increased sCD28 marker according to the presence of disease manifestations. Significant associations were demonstrated in neuropsychiatric lupus (OR 7.1 [1.8 – 67.9], p=0.047), nephritis (OR 14.5 [1.6 – 131.9], p=0.017), and mucocutaneous manifestations (OR 3.4 [1.9 – 12.8], p=0.035).

**Table 2.** Association of the soluble CD28 concentrations according to the clinical manifestations.

Clinical manifestations	Comparison analysis			Logistic regression analysis	
	Present (ng/ml)	Not present (ng/ml)	p	OR (95% CI)	p
Neuropsychiatric	28.9 ± 9.0	19.1 ± 9.9	0.031*	7.1 (1.8 – 67.9)	0.047*
Vasculitis	27.1 ± 8.5	20.1 ± 10.4	0.261	n/a	n/a
Arthritis	27.1 ± 14.1	19.4 ± 9.5	0.098	2.5 (0.5 – 15.8)	0.319
Myositis	24.7 ± 15.4	20.0 ± 9.6	0.348	1.8 (0.3 – 12.0)	0.553
Nephritis	24.8 ± 9.4	19.4 ± 10.4	0.043*	14.5 (1.6 – 131.9)	0.017*
Mucocutaneous	22.2 ± 9.2	19.4 ± 11.2	0.416	3.4 (1.9 – 12.8)	0.035*
Serositis	29.9 ± 12.9	18.6 ± 8.7	0.007*	3.4 (0.6 – 20.1)	0.178
Hematologic disorders	21.0 ± 10.4	20.2 ± 10.5	0.819	0.8 (0.2 – 2.9)	0.775

## Discussion

Systemic lupus erythematosus (SLE) is a complex autoimmune disorder that can impact multiple organs. The finding for markers to assess the disease activity in SLE is crucial for guiding treatment decisions, monitoring disease progression, evaluating treatment response, facilitating clinical trial design, and improving patient outcomes. These markers enable a more precise and personalized approach to the management of SLE, leading to better disease control, prevention of organ damage, and enhanced overall patient care. The role of CD28, a costimulatory molecule expressed on the surface of T-cells, has been an issue in evaluating disease activity in SLE. ^18^^–^^20^

This study showed a significant decrease in the expression of the CD28 surface marker among patients diagnosed with SLE, particularly in those with active SLE, when compared to healthy participants. Based on previous observations in patients with SLE have revealed a reduction in CD28 surface marker expression on T-cells, which has been linked to both disease activity and severity. ^20^^,^^21^ Similar to the findings in this study, our previous data indicated a correlation between the loss of CD28 surface markers on T-cells and the occurrence of SLE morbidities. ^8^ Although multiple studies have documented the association of CD28 ^+^ T-cells with SLE disease activity, there is a few detailed comparative studies for the CD28 ^+^ T-cell subsets in SLE. Minning *et al.* described that the CD8 ^+^CD28 ^+^ T-cells subset was reduced and inversely correlated with SLEDAI score. ^22^ Another study also showed that the peripheral CD4 ^+^ or CD8 ^+^ with CD28 ^+^ was negatively correlated with the CD28 ^+^ T-cells. ^20^ The functional characteristics of CD28 ^+^ T-cells exhibited increased production of Th1 proinflammatory cytokines along with cytotoxic molecules, perforin, and granzymes. ^23^ Therefore, these proinflammatory phenotypes might potentially impact the disease activity in SLE patients.

Compared to the CD28 surface marker, the levels of soluble CD28 (sCD28) were elevated in SLE patients and showed a positive correlation with disease activity. We also observed that the sCD28 had a better performance in predicting the active SLE patients compared to the surface CD28 marker or the conventional anti-dsDNA. Several specific clinical manifestations in SLE are also associated with the increased sCD28 concentration. The increase of sCD28 had been documented in SLE or other autoimmune diseases from previous studies. ^11^^,^^12^ Despite that, few studies had been demonstrated the performance of this marker for the monitoring of SLE disease activity. ^19^

The correlation between the CD28 surface marker and the CD28 soluble marker is complex. Under certain conditions, CD28 can be cleaved from the T-cell surface, releasing sCD28 into the bloodstream. The soluble CD28 can be generated via the membrane form being shed or by alternative mRNA splicing. ^24^ The elevated shedding of sCD28 could indicate high T-cell activity in SLE patients, resulting in higher levels of sCD28 in the serum. In contrast to the soluble form of CD28, the chronic activation and dysregulation of T-cells among SLE patients might lead to T-cell exhaustion. ^25^ The T-cell exhaustion was linked to the disappearance of CD28 expression on the T-cell surface, which was substituted by inhibitory molecules like programmed cell death 1 (PD-1). ^26^ The association between the CD28 molecules with the disease activity might represent the dysregulation of the T-cell response in SLE. This finding was also confirmed by the previous analysis that the T-cell activation dysregulations had the clinical implications in SLE patients. ^27^

This study has certain limitations, as it employs a cross-sectional design, potentially resulting in a weak casual-effect relationships. Therefore, a longitudinal study in the future will provide a better insight into the association between the CD28 markers with the disease activity, risk for relapse, or therapeutic efficacy. Although the sample of this study was relatively low, we believed that the group of patients selected was the most suitable for conducting this analysis. The patients and healthy individuals were carefully selected, and the identification of disease activity and clinical manifestations were examined accordingly.

In conclusion, our findings confirm that CD28 markers are indeed linked to disease activity in SLE. Some of the clinical manifestations are also associated with the levels of CD28 markers. In general, this study indicates a potential role of CD28 in predicting disease activity in SLE. However, this current study requires further research through longitudinal studies to provide multiple advantages in assessing disease activity, personalized treatment strategies, prognostic value, mechanistic insights, and potential therapeutic targets. As research in this field advances, these benefits could contribute to improving patient care and a more profound understanding of the complex mechanisms underlying SLE pathogenesis.

## Data Availability

Figshare: Role of Soluble CD28 as the Predictor of Disease Activity in SLE, https://doi.org/10.6084/m9.figshare.23897154. ^28^ Data are available under the terms of the Creative Commons Zero “No rights reserved” data waiver (CC0 1.0 Public domain dedication).
