# Supplementary material for: Association of the CD28 markers with the disease activity in systemic lupus erythematosus patients
Source: F1000Res. 2023 Oct 18;12:1362. [Version 1] doi: 10.12688/f1000research.140890.1 (PMC12514439; doi:10.12688/f1000research.140890.1)
Supplement: Peer review history

# Reviewer report: Reviewer response for version 1

**DOI:** 10.5256/f1000research.154295.r413825

**Published:** 2025-10-10

**Authors:** Angel Justiz-Vaillant

**Affiliations:** 1 Eric Williams Medical Science Complex, The University of the West Indies at St Augustine, Saint Augustine, Tunapuna/Piarco Municipal Corporation, Trinidad and Tobago

This study investigates the role of CD28 markers in assessing disease activity and clinical manifestations in Systemic Lupus Erythematosus (SLE). The research compares CD28 marker levels in 40 female SLE patients (divided into active SLE and low disease activity state, LLDAS groups) and 20 healthy female controls. The study employs flow cytometry and ELISA assays to measure CD28 expression on T-cells and soluble CD28 (sCD28) concentrations in serum.

 The findings indicate that active SLE patients exhibit lower percentages of CD4+CD28+ T-cells but higher concentrations of sCD28 compared to LLDAS patients and healthy controls. A moderate positive correlation was observed between sCD28 concentration and SLE disease activity. Higher sCD28 concentrations were associated with specific clinical manifestations, including neuropsychiatric lupus, nephritis, and mucocutaneous manifestations.

 ROC curve analysis suggests that sCD28 demonstrates potential as a biomarker for distinguishing active SLE from LLDAS, with a higher AUC score compared to CD28 surface markers and anti-dsDNA. The authors conclude that CD28 markers, particularly sCD28, are linked to SLE disease activity and clinical manifestations, suggesting a potential role in predicting disease activity. However, they emphasize the need for further longitudinal studies to validate these findings and explore the underlying mechanisms. This study contributes to the growing body of evidence supporting the use of biomarkers for improved diagnosis, assessment, and management of SLE.

Is the work clearly and accurately presented and does it cite the current literature?

Yes

If applicable, is the statistical analysis and its interpretation appropriate?

Yes

Are all the source data underlying the results available to ensure full reproducibility?

Yes

Is the study design appropriate and is the work technically sound?

Yes

Are the conclusions drawn adequately supported by the results?

Yes

Are sufficient details of methods and analysis provided to allow replication by others?

Yes

Reviewer Expertise:

Clinical Immunology

I confirm that I have read this submission and believe that I have an appropriate level of expertise to confirm that it is of an acceptable scientific standard.

## References

[1] : Imbalance between CD8CD28and CD8CD28T-cell subsets and its clinical significance in patients with systemic lupus erythematosus. *Lupus* .2019;28(10) : 10.1177/0961203319867130 1214-1223 10.1177/0961203319867130 31399013

# Reviewer report: Reviewer response for version 1

**DOI:** 10.5256/f1000research.154295.r327707

**Published:** 2024-10-12

**Authors:** Reem Hamdy A. Mohammed

**Affiliations:** 1 Rheumatology Department, Faculty of Medicine, Cairo University, Giza, Egypt

The article presented illustrates the link between the CD28 expression on CD 4 and CD8 T cells and its' relation to disease activity and organ involvement in lupus. This is a case-control study with a cross sectional design though the study design wasn't mentioned yet the methodology illustrated the design clearly. The objective of the study is very interesting especially being displayed for the Indonesian population which increases the body of evidence in another population other than European and American data that are readily available. This study showed a significant decrease in the expression of the CD28 surface marker among patients diagnosed with SLE, particularly in those with active SLE, when compared to healthy participants. The study analysis  showed that the levels of soluble CD28 (sCD28) were elevated in SLE patients and showed a positive correlation with disease activity and correlated with neuropsychiatric, mucocutaneous and nephritis in the study group. The authors also observed that the CD28 had a better performance in predicting the active SLE patients compared to the surface CD28 marker or the conventional anti-dsDNA. The authors mentioned their study limitation and declared data availability with links provided.

Is the work clearly and accurately presented and does it cite the current literature?

Yes

If applicable, is the statistical analysis and its interpretation appropriate?

Yes

Are all the source data underlying the results available to ensure full reproducibility?

Yes

Is the study design appropriate and is the work technically sound?

Yes

Are the conclusions drawn adequately supported by the results?

Yes

Are sufficient details of methods and analysis provided to allow replication by others?

Yes

Reviewer Expertise:

Autoimmune disease and rheumatology.

I confirm that I have read this submission and believe that I have an appropriate level of expertise to confirm that it is of an acceptable scientific standard.

# Response

**Published:** 2024-10-14

**Authors:** Mirza Zaka Pratama

**Affiliations:** Rheumatology Division, Department of Internal Medicine, Faculty of Medicine Brawijaya University, Indonesia

Thank you for your thorough review and constructive feedback on our article. We appreciate your positive remarks about our study's objective, particularly the focus on the Indonesian population, which aims to broaden the understanding of systemic lupus erythematosus (SLE) beyond European and American contexts.

 We are glad that you found the methodology and statistical analysis appropriate, as well as the clarity in presenting the link between CD28 expression and disease activity in lupus. Additionally, we appreciate your acknowledgment of the significant findings, particularly the predictive performance of CD28 in active SLE cases compared to conventional markers like anti-dsDNA.

 We also take note of your observation about the study design not being explicitly mentioned. Although we believe the methodology section conveys the cross-sectional case-control nature of the study, we will incorporate an explicit statement about the design to enhance clarity for readers.

 Your feedback regarding the reproducibility of our data is encouraging, and we are pleased that our efforts to provide transparent access to the source data were well-received. If there are additional details you believe would further enhance the replicability of the study, we are happy to address them.

 Once again, we appreciate your valuable feedback and thoughtful insights, which have contributed to improving the quality of our work.
